# Bayesian Models of Individual Differences: Combining Autistic Traits and Sensory Thresholds to Predict Motion Perception

**DOI:** 10.1177/0956797616665351

**Published:** 2016-10-21

**Authors:** Georgie Powell, Zoe Meredith, Rebecca McMillin, Tom C. A. Freeman

**Affiliations:** School of Psychology, Cardiff University

**Keywords:** perception, eye movements, autism, motion perception, open data

## Abstract

According to Bayesian models, perception and cognition depend on the optimal combination of noisy incoming evidence with prior knowledge of the world. Individual differences in perception should therefore be jointly determined by a person’s sensitivity to incoming evidence and his or her prior expectations. It has been proposed that individuals with autism have flatter prior distributions than do nonautistic individuals, which suggests that prior variance is linked to the degree of autistic traits in the general population. We tested this idea by studying how perceived speed changes during pursuit eye movement and at low contrast. We found that individual differences in these two motion phenomena were predicted by differences in thresholds and autistic traits when combined in a quantitative Bayesian model. Our findings therefore support the flatter-prior hypothesis and suggest that individual differences in prior expectations are more systematic than previously thought. In order to be revealed, however, individual differences in sensitivity must also be taken into account.

Bayesian models have become popular means of explaining behavior in a number of psychological domains. These behaviors range from how people judge basic perceptual features, such as motion, shape, orientation, and depth (Adams & Mamassian, 2004; Burge, Fowlkes, & Banks, 2010; Girshick, Landy, & Simoncelli, 2011; Weiss, Simoncelli, & Adelson, 2002), to the cognitive and motor decisions people make following perceptual processing (Chater & Oaksford, 2008; Griffiths, Kemp, & Tenenbaum, 2008). Bayesian models are based on the assumption that perceptual and cognitive choices arise from a statistically optimal combination of noisy incoming evidence and prior knowledge (Ernst & Bülthoff, 2004; Knill & Pouget, 2004; Vilares & Kording, 2011). They formalize an intuitive central tenet: As evidence becomes less reliable, people are increasingly influenced by prior expectations. This simple idea explains why visual stimulation under ambiguous conditions can lead to perceptual illusions and biases (e.g., Powell, Bompas, & Sumner, 2012) and why linguistic probabilities influence the interpretation of ambiguous sentences (Chater & Manning, 2006). Bayesian frameworks have also been used to explain features of clinical conditions such as autism spectrum disorder (ASD; Pellicano & Burr, 2012), schizophrenia (Fletcher & Frith, 2009; Teufel et al., 2015), and anxiety and depression (Paulus & Yu, 2012). Given that these disorders lie on spectra that extend to the general population (e.g., Baron-Cohen, Wheelwright, Skinner, Martin, & Clubley, 2001), Bayesian models may also capture more fine-grained differences across people in a systematic fashion. We therefore investigated whether individual differences in perceived motion are determined by predictable differences in prior expectation once differences in sensory sensitivity have been taken into account. To do this, we tested a quantitative model inspired by Pellicano and Burr’s (2012) Bayesian explanation of autism.

In Experiment 1, we investigated the lowering of perceived speed during pursuit eye movements (the *Aubert-Fleischl phenomenon*), and in Experiment 2, we investigated the lowering of perceived speed as contrast is reduced (Thompson, 1982). In Bayesian models of phenomena such as these, likelihood functions and probability distributions are used to encapsulate noisy sensory evidence and prior expectations, respectively. For motion, the prior for speed peaks at zero, which reflects the fact that most objects are at rest or move slowly (Weiss et al., 2002). Likelihood and prior are multiplied together to yield a posterior distribution, the average of which forms the basis of perceptual judgments, such as estimating speed (Fig. 1, left- and right-hand panels). As sensory evidence becomes less reliable, the likelihood distribution spreads out, which shifts the posterior distribution toward the prior and lowers the speed estimate. Low-contrast stimuli therefore appear to move more slowly, because reducing the contrast makes the sensory evidence less reliable, which allows the prior to dominate the estimate of speed (Hürlimann, Kiper, & Carandini, 2002; Sotiropoulos, Seitz, & Seriès, 2014; Stocker & Simoncelli, 2006, although see Hassan & Hammett, 2015). Similarly, in the case of the Aubert-Fleischl phenomenon, pursuit eye movements lower the reliability of sensory evidence, which causes motion thresholds to increase along with a corresponding slowing of perceived speed (Freeman, Champion, & Warren, 2010).

**Fig. 1.**
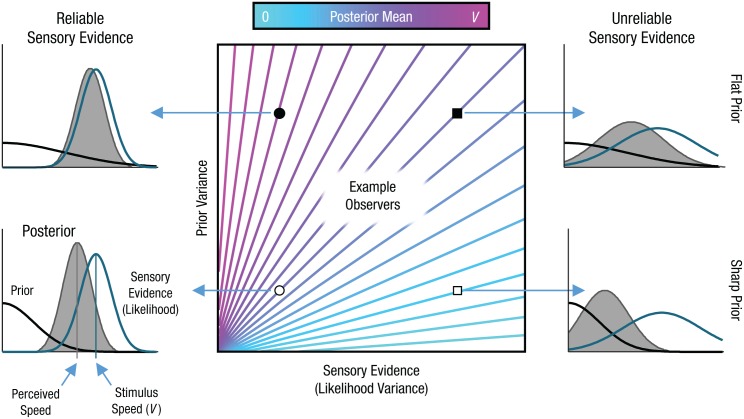
Schematic of the Bayes model of speed perception. The central panel shows how the variance of the prior distribution and the sensory evidence influence perceived speed (mean of the posterior distribution, color-coded from 0 to stimulus speed *V*). The surrounding panels depict specific examples of how these contours were derived; each contour corresponds to an observer (identified by symbols in the central panel) with a particular combination of prior variance and level of sensory evidence. The top two panels have the same (flatter) prior but the sensory evidence (likelihood) becomes less reliable from left to right; the bottom two panels have the same sharper priors. In each case, the statistically optimal best guess is based on multiplying the sensory evidence (likelihood function) with the prior distribution to yield the posterior distribution. As the sensory evidence gets less reliable (cf. left and right panels), the location of the posterior distribution shifts toward the prior distribution, so perceived speed goes down. Similarly, as the prior distribution becomes flatter (cf. bottom and top panels on each side), the location of the posterior distribution shifts toward the sensory evidence, so perceived speed goes up. The general relationship between the location (mean) of the posterior and the variance of prior distribution and sensory evidence is shown in the central panel. Assuming distributions are Gaussian, that observers are unbiased (i.e., their likelihood mean equals stimulus speed *V*, as shown in the lower-left panel), and that the prior is centered on zero, then the posterior’s location is determined by the ratio of the variances. This is shown as color-coded iso-mean contours. Each contour therefore defines a line of constant perceived speed.

Two ways to manipulate sensory noise within an individual are by changing the contrast of a stimulus and inducing eye movements. But noise can also vary across individuals, which implies that the reliability of sensory evidence also differs from person to person. This leads to differences in psychophysical thresholds, some of which have recently been related to changes in structural, neuropharmacological, and electrophysiological factors (Edden, Muthukumaraswamy, Freeman, & Singh, 2009; Song, Schwarzkopf, Kanai, & Rees, 2015). At the same time, priors are also known to vary between people (Adams, 2007). In particular, the shapes of priors for speed seem to be unique to individuals when reverse-engineered from psychophysical data (Sotiropoulos et al., 2014; Stocker & Simoncelli, 2006). Yet reasons why priors differ across individuals have received less attention than reasons why the reliability of sensory evidence might change.

We therefore investigated whether individual differences in motion priors are more systematic than previously thought. We did this by testing a model designed to account for a particular aspect of the shape of the prior, namely its variance. The model was based on the ideas of Pellicano and Burr (2012), who suggested that priors are flatter in individuals with ASD than in individuals without ASD. The *flatter-prior* hypothesis could explain a number of features of autism, such as resistance to certain visual illusions (see Dakin & Frith, 2005; Simmons et al., 2009), sensory overload, and insistence on sameness. Initial support for the flatter-prior hypothesis comes from Skewes, Jegindø, and Gebauer (2015), who found that a group scoring high on an autism-trait questionnaire was less able to learn short-term prior information than a group scoring lower on the same questionnaire when the proportion of visual targets embedded in a detection task was manipulated. Similarly, Zaidel, Goin-Kochel, and Angelaki (2015) found that individuals with ASD failed to learn changes in stimulus reliability when noise was added to visual motion in a task in which observers judged the direction of their self-motion. From a computational standpoint, Rosenberg, Patterson, and Angelaki (2015) have shown how changes to divisive normalization in populations of neurons could lead to flatter priors in autism.

If the shapes of priors vary as a function of the degree of autistic traits in the general population, then demonstrating a relationship between prior distributions and the experience of illusions would be difficult because the reliability of sensory evidence could also differ across individuals. This is demonstrated in the central panel of Figure 1, where the color-coded contours define the position of the posterior distribution from which perceptual estimates arise. According to the Bayesian framework, any two individuals on a contour (e.g., those represented in Fig. 1 by an open circle and a filled square) should make identical perceptual judgments despite very different priors and likelihoods. In comparison, individuals who differ in only their prior or likelihood (e.g., circles and squares; open and filled symbols) should make different perceptual judgments. It is therefore impossible to predict individual differences in dimensions such as perceived speed by examining only differences in prior or sensitivity: Both must be taken into account.

To investigate whether Pellicano and Burr’s (2012) flatter-prior hypothesis can account for individual differences in motion perception, we therefore incorporated an autism-trait measure into a formal Bayesian model. Crucially, the model also included threshold data to encapsulate the reliability of sensory evidence. We then used the model to predict individual differences in the strength of the Aubert-Fleischl phenomenon (Experiment 1) and the contrast effect (Experiment 2), comparing the results to changes in perceived speed measured in the lab.

## Method

### Experiment 1

#### Stimuli and materials

**Fig. 2.**
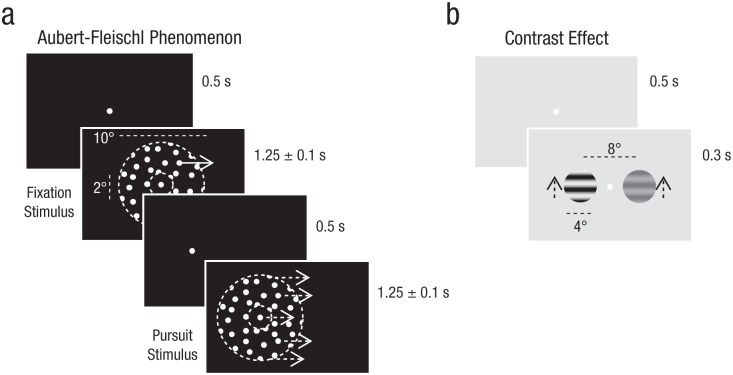
Example stimulus sequences used to assess (a) the strength of the Aubert-Fleischl phenomenon (Experiment 1) and (b) the size of the contrast effect (Experiment 2). In each trial of Experiment 1, participants viewed two sequentially presented random dot patterns, each of which moved inside an annulus window (indicated here with dotted lines that did not appear in the actual stimulus display). In fixation displays (in which the fixation point and annulus window were stationary), the dots appeared to move as a rigid sheet within the window, whereas in pursuit displays, the dots, window, and fixation point all moved together horizontally at the same velocity across the screen. The order of stimulus presentation and direction of stimulus movement was randomized across trials. In each trial of Experiment 2, participants viewed two vertically moving gratings presented simultaneously; both gratings were the same except that one was high contrast and one was low contrast. Movement direction (up vs. down) was chosen randomly on each trial. In both experiments, observers had to choose which of the two stimuli appeared to move faster.

The Aubert-Fleischl phenomenon was investigated using a two-alternative forced-choice technique consisting of two moving stimuli (A and B) combined in three different ways to create three different trial types (A-A, B-B, and A-B). Stimulus A was a patch of randomly positioned dots (0.64 dots/deg^2^, dot diameter = 0.1°) that moved across the screen relative to a stationary fixation point, and stimulus B was a moving patch that observers followed across the screen with a smooth-pursuit eye movement. Each stimulus was shown for a randomly selected duration between 1.15 and 1.35 s and was preceded by a solitary stationary fixation point presented for 0.5 s (see Fig. 2a for an example of an A-B trial sequence). The random dot patterns were displayed through an annulus window (inner diameter = 2°, outer diameter = 10°) whose motion was yoked to the motion of the central fixation point. For stimulus A, the dot pattern therefore appeared to move as a rigid sheet behind a static annulus window. For stimulus B, the dots, annulus window, and fixation point all moved at the same velocity. Stimulus motion was always horizontal, with direction (left vs. right) chosen at random on each trial.

Stimuli were viewed on a ViewSonic P225f monitor at a frame rate of 100 Hz in a completely darkened lab at a distance of 70 cm. Eye movements were recorded using an EyeLink 1000 video-based eye tracker (SR Research, Kanata, Ontario, Canada) that sampled eye position at a rate of 1000 Hz. Autistic traits were measured with the Autism-Spectrum Quotient questionnaire (Baron-Cohen et al., 2001). The questionnaire consists of 50 questions, and scores range from 0 (no autistic traits) to 50 (high level of autistic traits).

#### Procedure

Two of the three different stimulus combinations (A-A and B-B) were used to measure speed-discrimination thresholds for fixated stimuli and pursued stimuli, respectively. The third combination (A-B) was used to measure the strength of the Aubert-Fleischl phenomenon, namely, the perceived speed difference between fixated stimuli and pursued stimuli. In discrimination trials, one of the stimuli was designated as the standard and had a fixed speed of 8° per second. For the Aubert-Fleischl trials, the pursued stimulus was designated as the standard and moved at a speed, *V_B_*, of 8° per second. Psychometric functions for all trial types were then obtained by manipulating the speed of the other stimulus (i.e., the test stimulus) across trials, according to a method of constant stimuli. Observers had to choose which of the two stimuli appeared to move faster. No feedback was given. The order of test and standard stimuli was randomized across trials.

Test stimuli were shown at seven speeds equally spaced 1.33° per second apart. Stimuli were shown at each speed 10 times for each trial type, which yielded 210 trials per observer. The speeds ranged from 4 to 12° per second for discrimination trials and 0 to 8° per second for Aubert-Fleischl trials. Both stimulus A and stimulus B were shown for approximately 1.25 s (*SD* = 0.1 s), with blank intervals of 0.5 s between them. All speeds and trial types were randomly intermixed within each testing session.

The autism-trait questionnaire was completed following the psychophysics measurements. Testing sessions lasted for about 30 min. Prior to data collection, the eye tracker was calibrated for each observer using standard procedures.

#### Data analysis

##### Psychophysics measurements

Dependent measures were obtained by fitting psychometric functions (cumulative Gaussians) to the appropriate subset of trials using probit analysis (examples can be found in Fig. S1 of the Supplemental Material available online). For A-B trials, the strength of the Aubert-Fleischl phenomenon was defined as the speed at which the test stimulus was chosen 50% of the time (i.e., the point of subjective equality, or PSE). The PSE therefore corresponded to the speed of the dots viewed with stationary fixation (*V_A_*) that produced a perceived-speed match to the pursued stimuli. For convenience (and in keeping with the formal model derivation shown in the Supplemental Material), we express the PSE as the ratio *V_A_*/*V_B_* (i.e., PSE/standard speed). Assuming that pursued dots appear slower to the observer, then the ratio would be less than 1. Thresholds for A-A and B-B discrimination trials were defined as the standard deviation of the best-fitting cumulative Gaussian function. This is equivalent to the speed difference between the 84.1% point and the PSE.

##### Eye movements

The accuracy of smooth pursuit was assessed using the mean eye speed across all stimulus-B presentations, regardless of trial type. This was converted into a gain measure by dividing by the standard speed (*V_B_* = 8°/s). Fixation accuracy was assessed using mean eye speed across all stimulus-A presentations, again regardless of trial type. All speed measures were based on the temporal derivative of smoothed positioned samples (Gaussian filter, *SD* = 16 Hz) with blinks, dropouts, and saccades removed using standard procedures.

#### Observers

All observers gave informed consent, and the experimental procedures were approved by the School of Psychology, Cardiff University Ethics Committee. Observers had normal or corrected-to-normal vision. Thirty-seven undergraduate students participated for course credit or payment (1 additional observer was unable to complete testing). One observer was excluded from the analysis because her responses in the Aubert-Fleischl trials did not exceed 50% correct (i.e., the PSE) regardless of stimulus speed. Four observers were removed because they failed to obey the eye movement instructions (2 moved their eyes substantially in the fixation intervals, with a mean velocity > 2°/s; 2 did not pursue properly, with a mean pursuit gain < 0.6). An additional 2 observers were excluded because of technical problems with the eye movement recordings (> 50% samples were lost because of eyeblinks and dropouts). This left a cohort of 31 observers (17 male, 14 female), with a mean age of 19.8 years (*SD* = 1.7). Our sample size was based on previous work in this area. The final sample was larger than the samples in the majority of the studies on individual differences cited in this article.

### Experiment 2

#### Stimuli and materials

The contrast effect and associated discrimination thresholds were investigated using a technique similar to that used in Experiment 1. Stimulus A was defined as a moving luminance grating (1 cycle per degree) shown at a high contrast (64%) through a circular window (diameter = 4°). Stimulus B was an identical grating displayed at a lower contrast (8%; see Fig. 2b for an example A-B trial sequence). Stimuli were displayed for 0.3 s and preceded by a stationary central fixation point displayed for 0.5 s. Unlike in Experiment 1, stimuli were shown simultaneously, and the motion was always vertical. The stimuli were positioned 4° to either side of the central fixation point, and direction (up vs. down) was chosen at random on each trial. Stimuli were viewed on a Sony Trinitron CPD-G400 monitor at a frame rate of 85 Hz in a completely darkened lab at a viewing distance of 57 cm. The mean luminance was 44.5 cd/m^2^, and the display was gamma corrected. Autistic traits were measured as in Experiment 1.

#### Procedure

For discrimination trials (A-A and B-B), the standard stimulus moved at a speed of 2° per second. For contrast-effect trials (A-B, as shown in Fig. 2b), the high-contrast stimulus was designated as the standard and moved at a speed, *V_A_*, of 2° per second. The standard was randomly selected to appear either on the left or the right of the screen. Psychometric functions for all three trial types were obtained using test speeds equally spaced 1° per second apart. Stimuli were shown at each speed 10 times for each trial type, which yielded 270 trials per observer. The speeds ranged from 1 to 4° per second for all trial types. As in Experiment 1, all speeds and trial types were randomly intermixed within each testing session.

#### Data analysis

The strength of the contrast effect and discrimination thresholds for high- and low-contrast stimuli were obtained using the same methods as Experiment 1. The only difference was that the standard was now defined as stimulus A. Hence the ratio *V_A_*/*V_B_* for the contrast effect corresponded to the speed of the standard stimulus divided by the PSE. Assuming that lower-contrast gratings appear slower, this ratio will be less than 1.

#### Observers

Twenty-eight undergraduate participated for course credit or payment. One observer was excluded from the analysis because her responses in the contrast-effect trials did not exceed 50% correct regardless of stimulus speed. Another was excluded because her PSE for the contrast effect was 15 standard deviations above the mean. This left a cohort of 26 observers (9 male, 17 female), with a mean age of 21.8 years (*SD* = 3.0). As in Experiment 1, our sample size was based on previous work in this area. The sample size in Experiment 2 was somewhere in the middle of the sample sizes in the majority of the studies on individual differences cited in this article.

### Model and analysis

The algebraic derivation of the model is described more fully in the Supplemental Material. The model is based on the assumption of an unbiased observer (i.e., mean of sensory evidence equals stimulus speed) and a slow-motion prior (i.e., peak of the prior is 0), both common practice in the motion literature. The model is based on three assumptions: (a) the variance of the sensory evidence is fixed with respect to stimulus speed; (b) the estimates of speed for pursued and fixated dot patches share a common prior, as do those for high- and low-contrast gratings; and (c) the prior’s variance is linearly related to the autism-trait measure by a single scaling factor. These three assumptions are justified as follows. The fixed-noise assumption suffices for the small range of speeds that our method probed; it is equivalent to the near-universal practice of fitting a single cumulative Gaussian function to psychophysical data in order to obtain the psychometric function. The common prior assumption in the case of the Aubert-Fleischl phenomenon is based on the idea that the knowledge to be encapsulated by the prior concerns the motion of objects in the world, not pursuit or fixation (Freeman et al., 2010); this idea also applies to stimuli displayed at different contrasts (whether the same prior is used across all motion tasks is beyond the scope of the current article). Finally, the assumption that the variance of the prior is linearly related to the autism trait was, in effect, tested in our experiments.

To model individual differences in the strength of the Aubert-Fleischl phenomenon or the contrast effect, we combined the preceding assumptions with standard Bayesian formulae and signal detection theory. This yielded the following equation (see the Supplemental Material for the derivation):

$$\frac{V_{A}}{V_{B}}=\frac{kQ+\Delta_{A}^{2}/2}{kQ+\Delta_{B}^{2}/2}.$$

The ratio *V_A_*/*V_B_* defines the size of the Aubert-Fleischl phenomenon (Experiment 1) or contrast effect (Experiment 2); ∆^2^A is the squared threshold for fixated or high-contrast stimuli, and ∆^2^B is the squared threshold for pursued or low-contrast stimuli; *Q* is the autism quotient; and *k* is the linear scaling factor. Note that the addition of prior and likelihood variances in the above equation (i.e., kQ + ∆^2^ /2) should not be confused with the multiplication of prior distributions with likelihood functions. It is the latter that lies at the core of the derivation.

To investigate the model, we determined the single value of *k* that minimized the squared error between the actual size of the Aubert-Fleischl phenomenon or contrast effect and those predicted by the model using the above equation. There was therefore a single value of *k* per cohort of observers. We then compared the goodness of fit of the Bayes model to two reduced and non-Bayesian versions. The first ignored the sensory evidence and included the prior only, using the best-fitting value of *k* obtained from the autism quotient without the thresholds (i.e., *V_A_*/*V_B_* = *kQ*). The second model, based on the ratio of the squared thresholds alone, ignored the prior and used only the sensory evidence. For consistency, we also allowed the threshold ratio to be scaled by its own best-fitting value of *k* (i.e., VAVB = k∆^2^A /∆^2^B ). Hence all three models had a single free parameter.

## Results

**Fig. 3.**
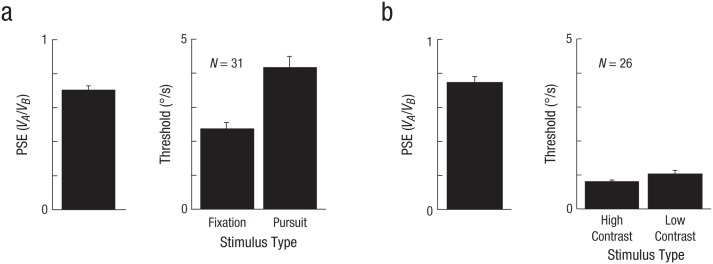
Mean point of subjective equality (PSE) and mean discrimination thresholds for (a) Experiment 1, which investigated the Aubert-Fleischl phenomenon, and (b) Experiment 2, which investigated the contrast effect. Discrimination thresholds are shown separately for each stimulus type. The PSE was defined as the speed at which participants indicated that the test stimulus was faster than the standard stimulus 50% of the time. Error bars show +1 *SE*.

At a group level, both sets of observers had autism-trait scores close to previously published normative values (Experiment 1: *M* = 15.6, *SD* = 7.7; Experiment 2: *M* = 15.2, *SD* = 8.1; cf. the findings of Baron-Cohen et al., 2001: *M* = 16.4, *SD* = 6.3; also see trait vs. threshold scatterplots in Fig. S2 in the Supplemental Material). Before examining individual differences, we looked to see whether observers on average exhibited the expected lowering of perceived speed (Aubert-Fleischl phenomenon and contrast effect) and appropriate differences in thresholds (pursuit > fixation in Experiment 1, and low contrast > high contrast in Experiment 2). Figure 3 shows the group effects for the psychophysical measurements. For both experiments, the mean ratio *V_A_*/*V_B_* that defined the motion phenomenon was significantly less than one. Experiment 1 therefore showed a significant Aubert-Fleischl phenomenon, one-sample *t*(30) = −12.20, *p* < .001; pursued stimuli appeared to move more slowly than fixated stimuli. Further, Experiment 2 showed a significant contrast effect, one-sample *t*(25) = −7.22, *p* < .001; low-contrast stimuli appeared to move more slowly than high-contrast stimuli. At a group level, therefore, the perceptual data replicated previous findings (Freeman et al., 2010; Hürlimann et al., 2002; Sotiropoulos et al., 2014; Stocker & Simoncelli, 2006; Thompson, 1982).

The respective threshold differences were also in the direction predicted by the Bayesian framework: Pursuit thresholds were significantly greater than fixation thresholds, *t*(30) = 4.97, *p* < .001, and low-contrast thresholds were significantly greater than high-contrast thresholds, *t*(25) = 2.68, *p* = .013. On average, therefore, the threshold data show that the sensory evidence for motion during pursuit and for lower contrasts is less reliable and so, according to Bayesian framework, culminates in a slowing of perceived speed. The critical question of interest, however, is whether the individual differences in thresholds can predict individual differences in the two motion phenomena when combined with systematic differences in the prior.

**Fig. 4.**
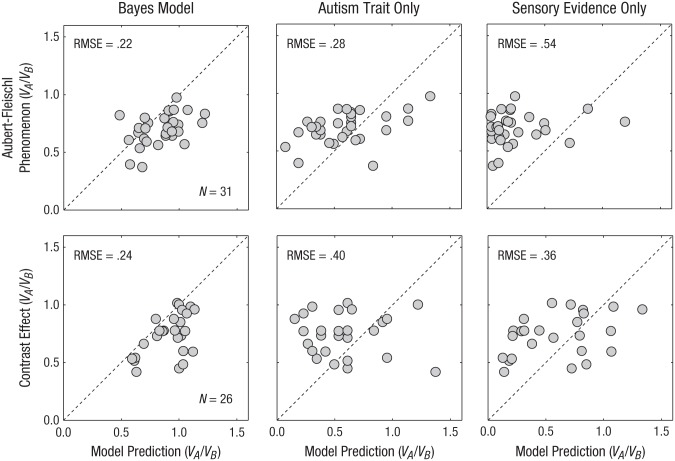
Scatterplots showing the size of the Aubert-Fleischl phenomenon (Experiment 1; top row) and the size of the contrast effect (Experiment 2; bottom row) as a function of the predictions of the Bayesian model (left panels), the reduced model with autism traits only (center panels), and the reduced model with sensory evidence only (right panels). For the Aubert-Fleischl phenomenon, the ratio *V_A_*/*V_B_* refers to the point of subjective equality (PSE) divided by the speed of the standard stimulus. The PSE was defined as the speed at which participants indicated that the test stimulus was faster than the standard stimulus 50% of the time. A value lower than 1 therefore means that a moving stimulus appeared slower when pursued than when fixated. For the contrast effect, however, the ratio *V_A_*/*V_B_* refers to the speed of the standard, high-contrast stimulus divided by the PSE. A value lower than 1 therefore means that low-contrast stimuli appeared to be slower than high-contrast stimuli. The dashed diagonal lines indicate perfect prediction. RMSE = root-mean-square error.

To examine this, we compared the size of each individuals’ measured Aubert-Fleischl phenomenon or contrast effect against the predictions made by the Bayesian model. The left column of Figure 4 shows the results for observers in each of the experiments. For comparison, reduced models based on either autism trait alone or sensory evidence alone are also shown (the distributions of traits and relative sensory reliability can be seen by recalling that the *x*-axes in these cases correspond to the trait scores and ratio of squared thresholds, respectively, both multiplied by a single scalar *k*). The dotted diagonal lines indicate a perfect fit. The Bayes model provided the best account of the data for both motion phenomena. The differences between models were captured by the root-mean-square errors (RMSEs) between predictions and data, the values of which are given in each panel. To investigate whether the numerical differences in RMSE were driven by outliers, we also calculated the mean absolute deviation, which avoids the squaring operation. In doing so, we found that the proportional differences between the Bayes model and reduced models actually increased.

The Bayes model is based on the assumption that the prior and sensory evidence are independent. To investigate this assumption, we first examined the four possible correlations between autism trait and thresholds (see Fig. S2 in the Supplemental Material for associated scatterplots). Three were nonsignificant (lowest *p* = .53), with Bayes factors (BFs) favoring the null hypothesis (lowest BF_01_ = 3.71;^1^ we assumed a flat prior for the correlation). The fourth correlation between autism trait and the low-contrast threshold was also nonsignificant (*p* = .07), but the Bayes factor for this case neither favored the null nor experimental hypothesis (BF_01_ = 0.86). For Experiment 1, we also looked at the correlations between the trait measure and eye movement accuracy, because any relationship between the two could act as an indirect link between the prior and the reliability of the sensory evidence. Neither correlation was significant (lowest *p* = .35). Our data therefore provide good support for key assumptions in the model.

In Experiment 1, the correlation between fixation threshold and fixation accuracy was significant, *r*(29) = −.38, *p* = .04. This potentially provides one reason why the sensory evidence associated with fixation varies across individuals. For instance, lower accuracy could mean an increase in fixation jitter, which implies that an observer was less able to hold his or her eyes stationary, so that greater external noise may be added to the stimulus motion (O’Connor, Margrain, & Freeman, 2010). Note that differences in fixation accuracy could not affect the results of Experiment 2 because the stimuli used to study the contrast effect were shown simultaneously.

In summary, our results provide support for Pellicano and Burr’s (2012) flatter-prior hypothesis, as long as individual differences in sensitivity are also taken into account. Our results further suggest that individual differences in the variance of the speed prior may be more systematic than previously thought.

## Discussion

We investigated whether individual differences in perception can be accounted for by a Bayesian model that combined individual differences in sensitivity with predictable individual differences in prior expectation. Our study was motivated by a need to unite two themes in the literature: a renewed interest in individual differences in cognitive neuroscience (e.g., Kanai & Rees, 2011) and mounting evidence that the Bayesian framework provides a principled way to describe perception and cognition (e.g., Vilares & Kording, 2011). According to the Bayesian framework, noisy incoming evidence and prior expectations are optimally combined. Hence, individual differences should be a function of both. A key feature of our model was the idea that certain features of the prior may vary from one person to another in a systematic way. Specifically, inspired by Pellicano and Burr’s (2012) flatter-prior hypothesis, we examined whether the variance of the motion prior was a function of autistic traits. These traits were combined with motion thresholds to predict individual differences in perceived speed using a quantitative Bayesian approach. The model fit the data well under two very different stimulus manipulations, one involving eye movement and the other involving contrast. Models based on autism traits or thresholds alone yielded much worse predictions.

One implication is that individual differences in prior and sensitivity could mask one another when perception between subpopulations is compared. This point is particularly relevant to the clinical literature (Dakin & Frith, 2005; Fletcher & Frith, 2009; Simmons et al., 2009; Teufel et al., 2015). For instance, in autism research, these differences have been traditionally attributed to either top-down processes (e.g., Happé & Frith, 2006) or bottom-up processes (e.g., Mottron, Dawson, Soulières, Hubert, & Burack, 2006). The Bayesian framework can unify these two traditions; however, our results suggest that individual differences in both must be taken into account. One person with autism might show a different pattern of behavior than another person with autism despite having the same prior, simply because the two individuals differ in sensitivity. There is no straightforward mapping between autism (or prior) and perception and cognition because the relationship is confounded by variation in sensory information, and vice versa. This point may also explain the divergent findings in the autism literature, such as those surrounding the debate on susceptibility to visual illusions (Chouinard, Unwin, Landry, & Sperandio, 2016; Simmons et al., 2009). But it also suggests that attributing the vagaries of perception in autism to either a flatter prior (Pellicano & Burr, 2012) or increased sensory precision (Brock, 2012) tells an incomplete story. Similar caution applies to other disorders to which Bayesian models have been applied (e.g., schizophrenia and depression) and to individual differences in general.

Our findings suggest that some of the between-subjects variation in priors may be more systematic than is implied in some formal Bayesian accounts. Specifically, the results suggests that the variance of the motion prior is influenced by the degree of autistic traits displayed by different individuals. However, this relationship is not necessarily exclusive—the model described here did not account for all the variance in the data, so other factors may contribute. For instance, we did not address any higher order aspects of the shape’s prior, just its spread; and we assumed that the likelihood is symmetric, an idea that has recently been challenged (Wei & Stocker, 2015). The model is also based on the assumption that the prior peaks at 0, a likely feature of the natural statistics of the motion world. But whether the internalized peak of the motion prior varies from person to person is an open question.

Why would sensory evidence and prior expectations differ across individuals? In terms of sensory evidence, these differences could be attributed to changes in the efficiency of neural coding, such as those known to exist in the tuning of low-level mechanisms (Edden et al., 2009; Song et al., 2015). In terms of the prior, these differences could be driven by individual differences in sensitivity. However, we found no evidence that thresholds correlated with the trait measures, a finding that supports the independence of prior and threshold at the heart of the model. Further support comes from Beierholm, Quartz, and Shams (2009), who showed that testing different contrasts delivered in sessions separated by a week did not affect estimates of priors, just the likelihoods. It is therefore more likely that individual differences in priors arise from other factors, such as different experiences of the world or the ability to learn from them. Information about the statistics of the world is gathered from many different sources, and people are likely to have different exposures to these. Moreover, some individuals may be less able than others to integrate and internalize the statistics of the world, an idea that lies at the heart of Pellicano and Burr’s (2012) hypothesis. It remains to be seen whether differences in lifelong acquisition of prior knowledge extend to more short-term learning, such as the autism-trait-mediated changes in criterion shifts found by Skewes et al. (2015) and the continual updating and error monitoring that underlie predictive coding theories (Lawson, Rees, & Friston, 2014; van Boxtel & Lu, 2013). It also remains to be seen whether the flatter-prior hypothesis applies to priors in all areas. For example, Pell et al. (2016) recently found that the prior for gaze direction remains intact in individuals with ASD.

In conclusion, we applied the popular Bayesian approach to the study of individual differences in perception. The results show that individual differences in priors and sensitivity must both be taken into account when explaining individual differences in perception. The underlying ideas presented here extend beyond perception and may inform the application of Bayesian models to cognition and psychopathologies.

## Supplementary Material

Supplementary material

Supplementary material

## Notes

1. The subscript 01 indicates that the BF reflects the extent to which the values indexed are more likely to be obtained under the null hypothesis (H_0_) than under the alternative hypothesis (H_1_).

## References

[bibr1-0956797616665351] Adams W. J. (2007). A common light-prior for visual search, shape, and reflectance judgments. Journal of Vision, 7(11), Article 11. doi:10.1167/7.11.11 17997666

[bibr2-0956797616665351] Adams W. J. Mamassian P. (2004). Bayesian combination of ambiguous shape cues. Journal of Vision, 4(10), Article 7. doi:10.1167/4.10.7 15595895

[bibr3-0956797616665351] Baron-Cohen S. Wheelwright S. Skinner R. Martin J. Clubley E. (2001). The autism-spectrum quotient (AQ): Evidence from Asperger syndrome/high-functioning autism, males and females, scientists and mathematicians. Journal of Autism and Developmental Disorders, 31, 5–17. 11439754 10.1023/a:1005653411471

[bibr4-0956797616665351] Beierholm U. R. Quartz S. R. Shams L. (2009). Bayesian priors are encoded independently from likelihoods in human multisensory perception. Journal of Vision, 9(5), Article 23. doi:10.1167/9.5.23 19757901

[bibr5-0956797616665351] Brock J. (2012). Alternative Bayesian accounts of autistic perception: Comment on Pellicano and Burr. Trends in Cognitive Sciences, 16, 573–574. 23123383 10.1016/j.tics.2012.10.005

[bibr6-0956797616665351] Burge J. Fowlkes C. C. Banks M. S. (2010). Natural-scene statistics predict how the figure–ground cue of convexity affects human depth perception. The Journal of Neuroscience, 30, 7269–7280. 20505093 10.1523/JNEUROSCI.5551-09.2010 PMC3062505

[bibr7-0956797616665351] Chater N. Manning C. D. (2006). Probabilistic models of language processing and acquisition. Trends in Cognitive Sciences, 10, 335–344. 16784883 10.1016/j.tics.2006.05.006

[bibr8-0956797616665351] Chater N. Oaksford M. (2008). The probabilistic mind: Prospects for Bayesian cognitive science. Oxford, England: Oxford University Press.

[bibr9-0956797616665351] Chouinard P. A. Unwin K. L. Landry O. Sperandio I. (2016). Susceptibility to optical illusions varies as a function of the autism-spectrum quotient but not in ways predicted by local-global biases. Journal of Autism and Developmental Disorders, 46, 2224–2239. doi:10.1007/s10803-016-2753-1 26914611

[bibr10-0956797616665351] Dakin S. Frith U. (2005). Vagaries of visual perception in autism. Neuron, 48, 497–507. 16269366 10.1016/j.neuron.2005.10.018

[bibr11-0956797616665351] Edden R. A. Muthukumaraswamy S. D. Freeman T. C. A. Singh K. D. (2009). Orientation discrimination performance is predicted by GABA concentration and gamma oscillation frequency in human primary visual cortex. The Journal of Neuroscience, 29, 15721–15726. 20016087 10.1523/JNEUROSCI.4426-09.2009 PMC6666191

[bibr12-0956797616665351] Ernst M. O. Bülthoff H. H. (2004). Merging the senses into a robust percept. Trends in Cognitive Sciences, 8, 162–169. 15050512 10.1016/j.tics.2004.02.002

[bibr13-0956797616665351] Fletcher P. C. Frith C. D. (2009). Perceiving is believing: A Bayesian approach to explaining the positive symptoms of schizophrenia. Nature Reviews Neuroscience, 10, 48–58. 19050712 10.1038/nrn2536

[bibr14-0956797616665351] Freeman T. C. A. Champion R. A. Warren P. A. (2010). A Bayesian model of perceived head-centered velocity during smooth pursuit eye movement. Current Biology, 20, 757–762. doi:10.1016/j.cub.2010.02.059 20399096 PMC2861164

[bibr15-0956797616665351] Girshick A. R. Landy M. S. Simoncelli E. P. (2011). Cardinal rules: Visual orientation perception reflects knowledge of environmental statistics. Nature Neuroscience, 14, 926–932. 21642976 10.1038/nn.2831 PMC3125404

[bibr16-0956797616665351] Griffiths T. L. Kemp C. Tenenbaum J. B. (2008). Bayesian models of cognition. In Sun R. (Ed.), The Cambridge handbook of computational psychology (pp. 59–100). Cambridge, England: Cambridge University Press.

[bibr17-0956797616665351] Happé F. Frith U. (2006). The weak coherence account: Detail-focused cognitive style in autism spectrum disorders. Journal of Autism and Developmental Disorders, 36, 5–25. 16450045 10.1007/s10803-005-0039-0

[bibr18-0956797616665351] Hassan O. Hammett S. T. (2015). Perceptual biases are inconsistent with Bayesian encoding of speed in the human visual system. Journal of Vision, 15(2), Article 9. doi:10.1167/15.2.9 25761348

[bibr19-0956797616665351] Hürlimann F. Kiper D. C. Carandini M. (2002). Testing the Bayesian model of perceived speed. Vision Research, 42, 2253–2257. 12220581 10.1016/s0042-6989(02)00119-0

[bibr20-0956797616665351] Kanai R. Rees G. (2011). The structural basis of inter-individual differences in human behaviour and cognition. Nature Reviews Neuroscience, 12, 231–242. 21407245 10.1038/nrn3000

[bibr21-0956797616665351] Knill D. C. Pouget A. (2004). The Bayesian brain: The role of uncertainty in neural coding and computation. Trends in Neurosciences, 27, 712–719. 15541511 10.1016/j.tins.2004.10.007

[bibr22-0956797616665351] Lawson R. P. Rees G. Friston K. J. (2014). An aberrant precision account of autism. Frontiers in Human Neuroscience, 8, Article 302. doi:10.3389/fnhum.2014.00302 PMC4030191 24860482

[bibr23-0956797616665351] Mottron L. Dawson M. Soulières I. Hubert B. Burack J. (2006). Enhanced perceptual functioning in autism: An update, and eight principles of autistic perception. Journal of Autism and Developmental Disorders, 36, 27–43. 16453071 10.1007/s10803-005-0040-7

[bibr24-0956797616665351] O’Connor E. Margrain T. H. Freeman T. C. (2010). Age, eye movement and motion discrimination. Vision Research, 50, 2588–2599. 20732343 10.1016/j.visres.2010.08.015

[bibr25-0956797616665351] Paulus M. P. Yu A. J. (2012). Emotion and decision-making: Affect-driven belief systems in anxiety and depression. Trends in Cognitive Sciences, 16, 476–483. doi:10.1016/j.tics.2012.07.009 22898207 PMC3446252

[bibr26-0956797616665351] Pell P. J. Mareschal I. Calder A. J. von dem Hagen E. A. H. Clifford C. W. G. Baron-Cohen S. Ewbank M. P. (2016). Intact priors for gaze direction in adults with high-functioning autism spectrum conditions. Molecular Autism, 7, Article 25. doi:10.1186/s13229-016-0085-9 PMC4832530 27087911

[bibr27-0956797616665351] Pellicano E. Burr D. C. (2012). When the world becomes too real: A Bayesian explanation of autistic perception. Trends in Cognitive Sciences, 16, 504–510. 22959875 10.1016/j.tics.2012.08.009

[bibr28-0956797616665351] Powell G. Bompas A. Sumner P. (2012). Making the incredible credible: Afterimages are modulated by contextual edges more than real stimuli. Journal of Vision, 12(10), Article 17. doi:10.1167/12.10.17 23024354

[bibr29-0956797616665351] Rosenberg A. Patterson J. S. Angelaki D. E. (2015). A computational perspective on autism. Proceedings of the National Academy of Sciences, USA, 112, 9158–9165. doi:10.1073/pnas.1510583112 PMC4522787 26170299

[bibr30-0956797616665351] Simmons D. R. Robertson A. E. McKay L. S. Toal E. McAleer P. Pollick F. E. (2009). Vision in autism spectrum disorders. Vision Research, 49, 2705–2739. 19682485 10.1016/j.visres.2009.08.005

[bibr31-0956797616665351] Skewes J. C. Jegindø E.-M. Gebauer L. (2015). Perceptual inference and autistic traits. Autism, 19, 301–307. doi:10.1177/1362361313519872 24523412

[bibr32-0956797616665351] Song C. Schwarzkopf D. S. Kanai R. Rees G. (2015). Neural population tuning links visual cortical anatomy to human visual perception. Neuron, 85, 641–656. 25619658 10.1016/j.neuron.2014.12.041 PMC4321887

[bibr33-0956797616665351] Sotiropoulos G. Seitz A. R. Seriès P. (2014). Contrast dependency and prior expectations in human speed perception. Vision Research, 97, 16–23. 24503425 10.1016/j.visres.2014.01.012 PMC4915944

[bibr34-0956797616665351] Stocker A. A. Simoncelli E. P. (2006). Noise characteristics and prior expectations in human visual speed perception. Nature Neuroscience, 9, 578–585. 16547513 10.1038/nn1669

[bibr35-0956797616665351] Teufel C. Subramaniam N. Dobler V. Perez J. Finnemann J. Mehta P. R., . . . Fletcher P. C. (2015). Shift toward prior knowledge confers a perceptual advantage in early psychosis and psychosis-prone healthy individuals. Proceedings of the National Academy of Sciences, USA, 112, 13401–13406. 10.1073/pnas.1503916112 PMC4629373 26460044

[bibr36-0956797616665351] Thompson P. (1982). Perceived rate of movement depends on contrast. Vision Research, 22, 377–380. 7090191 10.1016/0042-6989(82)90153-5

[bibr37-0956797616665351] van Boxtel J. J. Lu H (2013). A predictive coding perspective on autism spectrum disorders. Frontiers in Psychology, 4, Article 19. doi:10.3389/fpsyg.2013.00019 PMC3556598 23372559

[bibr38-0956797616665351] Vilares I. Kording K. (2011). Bayesian models: The structure of the world, uncertainty, behavior, and the brain. Annals of the New York Academy of Sciences, 1224, 22–39. doi:10.1111/j.1749-6632.2011.05965.x 21486294 PMC3079291

[bibr39-0956797616665351] Wei X.-X. Stocker A. A. (2015). A Bayesian observer model constrained by efficient coding can explain ‘anti-Bayesian’ percepts. Nature Neuroscience, 18, 1509–1517. 26343249 10.1038/nn.4105

[bibr40-0956797616665351] Weiss Y. Simoncelli E. P. Adelson E. H. (2002). Motion illusions as optimal percepts. Nature Neuroscience, 5, 598–604. 12021763 10.1038/nn0602-858

[bibr41-0956797616665351] Zaidel A. Goin-Kochel R. P. Angelaki D. E. (2015). Self-motion perception in autism is compromised by visual noise but integrated optimally across multiple senses. Proceedings of the National Academy of Sciences, USA, 112, 6461–6466. 10.1073/pnas.1506582112 PMC4443344 25941373

