# Supplementary material for: Bayesian Models of Individual Differences: Combining Autistic Traits and Sensory Thresholds to Predict Motion Perception
Source: Psychol Sci. 2016 Oct 21;27(12):1562–72. doi: 10.1177/0956797616665351 (PMC5367641; doi:10.1177/0956797616665351)
Supplement: Supplementary material [file Supplemental_Material.pdf]

Powell, Meredith, McMillin & Freeman

“Bayesian Models of Individual Differences: Combining Autistic Traits and Sensory Thresholds to Predict Motion Perception”

- Supplementary Material -

Derivation of the Bayes Model

The position (i.e. mean) of the posterior shifts from trial to trial due to noise at the measurement stage (for discussion, see Stocker & Simoncelli, 2006). The collection of posterior means therefore forms the distribution that underpins the psychometric function. This distribution has a mean (μ_S) and variance (σ_S^2) given by:

$$\mu_S = V \times \frac{\sigma_P^2}{\sigma_P^2+\sigma_L^2} \qquad (1)$$

$$\sigma_S^2 = \left(\frac{\sigma_P^2}{\sigma_P^2+\sigma_L^2}\right)^2 \times \sigma_L^2 \qquad (2)$$

where σ_P^2 and σ_L^2 are the variance of the prior and sensory evidence (likelihood), respectively (for derivation, see Freeman et al, 2010). Note that Eqn.(1) can be re-arranged to show that $V/\mu_S = 1 + \sigma_L^2/\sigma_P^2$, thus confirming that estimates of speed depend on the ratio of prior and likelihood variances as discussed in the main article.

For ‘bias trials’, the perceived speed of stimulus A (e.g. fixated or high contrast) and stimulus B (pursued of low contrast) are equal at the point of subjective equality (PSE). Hence the associated means of the posteriors that quantify the perceived speed for the two types of stimuli must be equal. From this identity and Eqn.(1):

$$\frac{V_A}{V_B} = \frac{\sigma_P^2+\sigma_L^2(V_A)}{\sigma_P^2+\sigma_L^2(V_B)} \qquad (3)$$

where $\sigma_L^2(V_A)$ and $\sigma_L^2(V_B)$ are constant according to the fixed-noise assumption (see Methods).

From Signal Detection Theory, discriminability (d’) is given by:

$$d' = \sqrt{2}\frac{\mu_1 - \mu_2}{(\sigma_1^2 + \sigma_2^2)^{1/2}} \qquad (4)$$

where ‘1’ and ‘2’ stand for the two intervals (either both fixation or both pursuit). Eqns. (1) and (2) provide the required terms in Eqn.(4):

$$\mu_1 - \mu_2 = (V_1 - V_1)\frac{\sigma_P^2}{\sigma_P^2 + \sigma_L^2} \qquad (5)$$

$$(\sigma_1^2 + \sigma_2^2)^{1/2} = \sqrt{2}\sigma_L \frac{\sigma_P^2}{\sigma_P^2 + \sigma_L^2} \qquad (6)$$

which, when substituted into Eqn.(4), gives:

$$d' = \frac{(V_1 - V_2)}{\sigma_L} \qquad (7)$$

The probability of choosing one interval over the other in a 2AFC task is $P = \Phi[d'/\sqrt{2}]$, where Φ is the cumulative function of the standard normal distribution. If threshold (Δ) is defined as the speed difference $(V_1 - V_2)$ at the 84.1% correct point on the psychometric functions, then $d' = \sqrt{2}$. Substituting into Eqn.(7) and rearranging:

$$\sigma_L^2 = \frac{1}{2}\Delta^2 \qquad (8)$$

From the linear-scaling assumption:

$$\sigma_P^2 = kQ \qquad (9)$$

where *Q* is the trait measure.

Substituting Eqns.(8) & (9) into (3) gives the Bayes model with one free parameter (*k*):

$$\frac{V_A}{V_B} = \frac{kQ + \Delta_A^2/2}{kQ + \Delta_B^2/2} \qquad (10)$$

Supplementary Figures

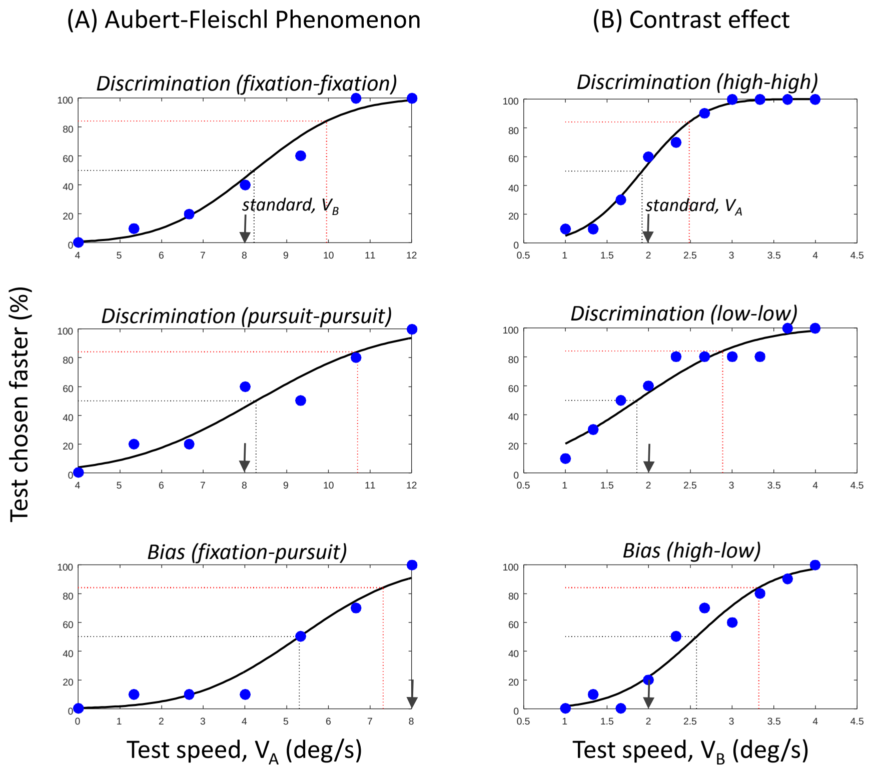

Figure S1: Example psychometric functions for (A) Aubert-Fleischl phenomenon (Experiment 1) and (B) the contrast effect (Experiment 2). The functions are based on a different representative observer from each experiment. The vertical arrow in each panel indicates the standard speed; the black dashed line shows the PSE and the red dashed line the 84.1% point. The top and middle rows correspond to discrimination trials. These were used to estimate the reliability of sensory evidence for speed perception during pursuit (top-left) and fixation (middle-left), or low contrast (top-right) and high contrast (middle right). The discrimination thresholds are equal to the difference in speed between red and black vertical dashed lines – the fact these are larger for the middle row compared to the top row indicates that the reliability of sensory evidence reduces during pursuit and low contrast for these observers. The bottom row shows the slowing of perceived speed during pursuit (bottom-left) and low contrast (bottom-right). This can be seen by noting the shift in the PSE (vertical black lines) away from the standard speed. It shifts to the left for the Aubert-Fleischl phenomenon because we chose the fixation stimulus to be the test in this case; hence its speed had to be decreased to match the pursued standard. The PSE shifts to the

right for the contrast effect because this time we chose the low-contrast stimulus to be the test; hence its speed has to be increased to match the high-contrast standard.

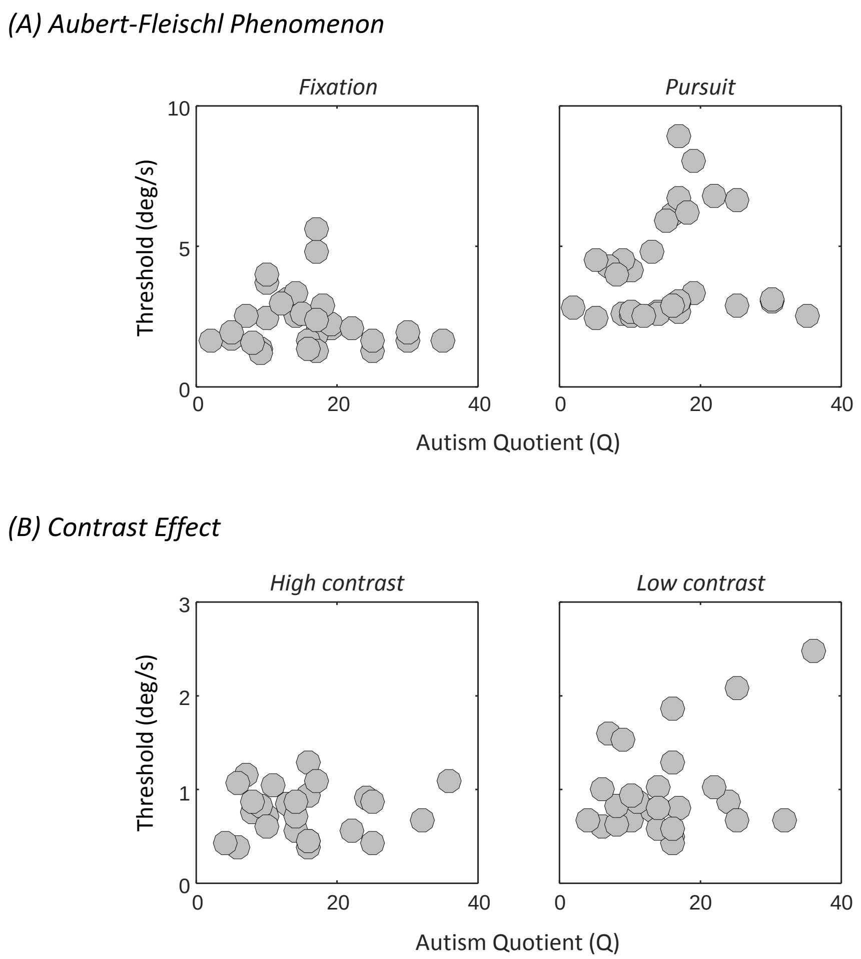

Figure S2: Scatterplots showing the lack of relationship between thresholds and autism trait for (A) Aubert-Fleischl phenomenon (Experiment 1) and (B) the contrast effect (Experiment 2).

References

Stocker, A. A., & Simoncelli, E. P. (2006). Noise characteristics and prior expectations in human visual speed perception. *Nature Neuroscience, 9*(4), 578-585.
