# Supplementary material for: Bayesian Models of Individual Differences: Combining Autistic Traits and Sensory Thresholds to Predict Motion Perception
Source: Psychol Sci. 2016 Oct 21;27(12):1562–72. doi: 10.1177/0956797616665351 (PMC5367641; doi:10.1177/0956797616665351)
Supplement: Supplementary material [file Open_Practices_Disclosure.pdf]

OPEN PRACTICES DISCLOSURE

PLEASE COMPLETE AND RETURN TO EDITORIALOFFICE@PSYCHOLOGICALSCIENCE.ORG

***Psychological Science* manuscript #:** **PSCI-16-0051.R1** **Corresponding author:** **Georgie Powell**

Articles accepted to *Psychological Science* after January 1, 2014, are eligible to earn badges that recognize open scientific practices: publicly available data, material, or preregistered research plans. Please read more about the badges on our Open Practices page, and you can also find information in the Open Science Framework wiki and FAQ.

☐ **Please check this box if you are not interested in participating.**

If you choose to participate, this form will be posted with your article as supplemental online material.

To apply for one or more badges acknowledging open practices, please check the appropriate box(es) below and provide the information requested in the relevant sections. You will not qualify for a badge for a given item unless you can provide a URL, doi, or other **permanent path** for accessing the specified information in a **public, open-access repository. Qualifying public, open-access repositories are committed to preserving data, materials, and/or registered analysis plans and keeping them publicly accessible via the web into perpetuity.** Examples include the Open Science Framework (OSF) and the various Dataverse networks. Hundreds of other qualifying data/materials repositories are listed at http://re3data.org/ and http://databib.org/. Preregistration of an analysis plan must take place via a publicly accessible registry system (e.g., OSF, ClinicalTrials.gov or other trial registries in the WHO Registry Network, institutional registration systems). **Personal websites and most departmental websites do not qualify as repositories.**

Authors who wish to publicly post third-party material in their data, materials, or preregistration plan must have the proper authority or permission agreement in order to do so.

There are circumstances in which it is not possible or advisable to share any or all data, materials, or a research plan publicly. For example, there are cases in which sharing participants' data could violate confidentiality. If you would like your article to include an explanation of such circumstances and/or provide links to any data or materials you have made available—even if not under conditions eligible to earn a badge—you may write an alternative note that will be published in the Open Practices note in the article. Please check this box if you would like your article to include an alternative note and provide the text of the note below:

☐ **Alternative Note:**

☑ **Application for Open Data Badge**

1. Provide the URL, doi, or other **permanent path** for accessing the data in a **public, open-access repository**:

   https://osf.io/m3neh/

   ☑ Confirm that there is sufficient information for an independent researcher to reproduce **all of the reported results**, including codebook if relevant.

☐ **Application for Open Materials Badge**

1. Provide the URL, doi, or other **permanent path** for accessing the materials in a **public, open-access repository**:

   ☐ Confirm that there is sufficient information for an independent researcher to reproduce **all of the reported methodology**.

☐ **Application for Preregistration Badge**

1. Provide the URL, doi, or other **permanent path** to the registration in a **public, open-access repository.***
2. Was the analysis plan registered prior to examination of the data or observing the outcomes? If no, explain.**
3. Were there additional registrations for the study other than the one reported? If yes, provide links and explain.*
4. Were there any changes to the preregistered analysis plan for the primary confirmatory analysis? If yes, explain.**
5. Are all of the analyses described in the registered plan reported in the article? If no, explain.*

*No badge will be awarded if (1) is not provided, **or** if (3) is answered "yes" without strong justification, **or** if (5) is answered "no" without strong justification.
**If the answer to (2) is "no," the notation DE (Data Exist) will be added to the badge, indicating that registration postdates realization of the outcomes but predates analysis. If the answer to (4) is "yes" with strong justification for changes, the notation TC (Transparent Changes) will be added to the badge, indicating that the analysis plan was altered but the preregistered analyses and rationale for the change are provided.

By signing below, authors affirm that the above information is accurate and complete, that any third-party material has been reproduced or otherwise made available only with the permission of the original author or copyright holder, and that publicly posted data do not contain information that would allow individuals to be identified without consent.

Name: [illegible] **Date:** **21/07/2016**
